# Machine learning and optical coherence tomography-derived radiomics analysis to predict persistent diabetic macular edema in patients undergoing anti-VEGF intravitreal therapy

**DOI:** 10.1186/s12967-024-05141-7

**Published:** 2024-04-16

**Authors:** Zhishang Meng, Yanzhu Chen, Haoyu Li, Yue Zhang, Xiaoxi Yao, Yongan Meng, Wen Shi, Youling Liang, Yuqian Hu, Dan Liu, Manyun Xie, Bin Yan, Jing Luo

**Affiliations:** 1 Department of Ophthalmology, The Second Xiangya Hospital, Central South University, 139 Middle Renmin Road, Changsha, 410011 China; 2 Department of Radiation Oncology, Shanghai East Hospital, School of Medicine, Tongji University, Shanghai, China; 3 Faculty of Infrastructure Engineering, Dalian University of Technology, Dalian, China; 4 University College London, London, UK

**Keywords:** Diabetic macular edema, OCT-omics, Anti-VEGF treatment response, Retinal imaging, Prognostic model

## Abstract

**Background:**

Diabetic macular edema (DME) is a leading cause of vision loss in patients with diabetes. This study aimed to develop and evaluate an OCT-omics prediction model for assessing anti-vascular endothelial growth factor (VEGF) treatment response in patients with DME.

**Methods:**

A retrospective analysis of 113 eyes from 82 patients with DME was conducted. Comprehensive feature engineering was applied to clinical and optical coherence tomography (OCT) data. Logistic regression, support vector machine (SVM), and backpropagation neural network (BPNN) classifiers were trained using a training set of 79 eyes, and evaluated on a test set of 34 eyes. Clinical implications of the OCT-omics prediction model were assessed by decision curve analysis. Performance metrics (sensitivity, specificity, F1 score, and AUC) were calculated.

**Results:**

The logistic, SVM, and BPNN classifiers demonstrated robust discriminative abilities in both the training and test sets. In the training set, the logistic classifier achieved a sensitivity of 0.904, specificity of 0.741, F1 score of 0.887, and AUC of 0.910. The SVM classifier showed a sensitivity of 0.923, specificity of 0.667, F1 score of 0.881, and AUC of 0.897. The BPNN classifier exhibited a sensitivity of 0.962, specificity of 0.926, F1 score of 0.962, and AUC of 0.982. Similar discriminative capabilities were maintained in the test set. The OCT-omics scores were significantly higher in the non-persistent DME group than in the persistent DME group (*p* < 0.001). OCT-omics scores were also positively correlated with the rate of decline in central subfield thickness after treatment (Pearson’s *R* = 0.44, *p* < 0.001).

**Conclusion:**

The developed OCT-omics model accurately assesses anti-VEGF treatment response in DME patients. The model’s robust performance and clinical implications highlight its utility as a non-invasive tool for personalized treatment prediction and retinal pathology assessment.

**Supplementary Information:**

The online version contains supplementary material available at 10.1186/s12967-024-05141-7.

## Introduction

Diabetic retinopathy (DR) is a common neurovascular complication of diabetes mellitus and a leading cause of visual impairment among working-age individuals worldwide [1]. Diabetic macular edema (DME) has emerged as the predominant form of vision-threatening DR, surpassing proliferative DR in terms of prevalence [2]. A comprehensive meta-analysis revealed a 6.8% global prevalence of DME in individuals aged 20–79 years with diabetes [3]. DME significantly impacts overall quality of life and imposes substantial socioeconomic burdens on healthcare systems.

Technological advancements, especially optical coherence tomography (OCT), have revolutionized DME management [4, 5]. OCT has emerged as a cornerstone imaging modality, enabling non-invasive retinal visualization and precise macular assessment [6, 7]. In recent years, anti-vascular endothelial growth factor (VEGF) intravitreal injections have emerged as a first-line treatment for DME [8], improving visual outcomes and altering DR’s management paradigm [9]. Notably, patients who respond favorably to initial treatment and experience a significant reduction in macular edema and improvement in visual acuity tend to have better long-term visual outcomes [10].

Multiple challenges exist in DME management and research. For instance, OCT-based central subfield thickness (CST), commonly employed as an endpoint in numerous clinical trials, inconsistently correlates with visual acuity [11]. OCT-derived prognostic markers like disorganisation of retinal inner layers (DRIL) show promise but are predominantly qualitative or semi-quantitative, limiting precision and reproducibility [12]. Additionally, approximately 40% of DME patients experience persistent DME (PDME) with minimal visual acuity and anatomical improvement despite undergoing multiple anti-VEGF therapies [13]. Notably, PDME lacks a consensus definition and management, leading to varying treatment approaches and outcomes [14]. The absence of standardized criteria for switching between anti-VEGF drugs or combining with corticosteroid therapy complicates treatment decision-making [15].

Radiomics, a valuable tool for high-throughput information extraction from medical images like computed tomography, magnetic resonance imaging, and ultrasound, has found applications in clinical diagnosis, prognosis, and treatment efficacy assessment in various disciplines, including oncology and diagnostics [16–18].

However, the application of radiomics in ophthalmology, particularly in OCT imaging, is relatively unexplored, Given the limitations in current markers for DME prognosis and management, our study aims to innovate by developing a machine learning predictive model that extracts radiomics features from OCT images, providing a quantitative, non-invasive solution that enhances DME management and assesses treatment efficacy.

## Methods

### Ethics

Ethical approval (No. 2017-053) for this retrospective study was obtained from the ethics committee of the Second Xiangya Hospital, Central South University. The study adhered to the Declaration of Helsinki and other applicable ethical guidelines. Patient data were anonymized and treated confidentially to ensure privacy and comply with data protection regulations.

### Patient cohort

Patients diagnosed with DME between May 2019 and January 2023 at the department of ophthalmology, the Second Xiangya Hospital of Central South University, were eligible for inclusion in this retrospective study. The medical records of eligible patients were retrospectively collected from the electronic medical record system.

The inclusion criteria were: age > 18 years; DME-related visual impairment and macular CST of ≥ 250 μm on OCT, where CST was determined as the mean thickness between the internal limiting membrane and Bruch’s membrane within the central 1-mm diameter of the Early Treatment Diabetic Retinopathy Study grid; receipt of ≥ 3 anti-VEGF intravitreal injections, including ranibizumab 0·5 mg/0·05 mL (Novartis Pharma AG, Basel, Switzerland), aflibercept 2·0 mg/0·05 mL (Bayer Healthcare Pharmaceuticals, Berlin, Germany), or conbercept 0·5 mg/0·05 mL (Kanghong Biotech Co., Ltd., Chengdu, China); and active follow-up prior to treatment and during the administration of at least the first three intravitreal injections. Exclusion criteria were: visual impairment from other ocular diseases (e.g., severe cataract, glaucoma, age-related macular degeneration, macular edema associated with retinal vein occlusion, vitreomacular traction, or uveitis); switch to intravitreal steroids or vitreoretinal surgery; and missing follow-up information, including poor quality of medical images. In this study, PDME was defined as any visual deterioration or inadequate CST reduction after three intravitreal anti-VEGF treatments. Specific CST reduction cut-offs based on recommendations by Sorour et al [14]: <10% for ≤ 400 μm (pre-treatment status, same as below), < 15% for 401–500 μm, < 20% for 501–600 μm, and < 25% for > 600 μm. All eyes were randomly divided into training and test sets at a ratio of 7:3.

### Imaging acquisition and region of interest segmentation

In this study, we introduced the concept of “OCT-omics,” a novel approach that combines “OCT” and “omics” to extract and analyze high-throughput quantitative features from OCT images, providing a comprehensive representation of retinal pathology.

Spectral-domain OCT images were acquired using the Optovue RTVue XR Avanti system (Optovue Inc., Fremont, CA, USA) for all eyes in this study. Follow-up assessments utilized radial scans (6 × 6 mm) centered on the fovea. Region of interest (ROI) segmentation and feature extraction were performed on DICOM-formatted B-scan OCT images along the vertical meridian.

Manual segmentation of inner retinal layers (from the inner limiting membrane to the external limiting membrane) [19, 20] was carried out using 3D Slicer software (version 5.0.3, https://www.slicer.org/) [21]. The segmentation was performed independently by a radiation oncologist (Y.C., 6 years of experience) and an ophthalmologist (Z.M., 5 years of experience) without knowledge of patient groups or treatment outcomes, ensuring objectivity and minimizing bias. The segmentation results were then reviewed and confirmed by a senior retina specialist (J.L., 30 years of experience).

### Feature extraction and selection

Radiomics features were extracted from the segmented images using the Pyradiomics module within the 3D Slicer software platform [22]; OCT images were resampled to voxel dimensions of 1 × 1 × 1 mm, and the bin width value was set at 25.0. The extracted features encompassed eight distinct classes: first-order statistics, shape features, texture features based on the gray-level run length matrix (GLRLM), gray-level co-occurrence matrix (GLCM), gray-level size zone matrix (GLSZM), gray-level dependence matrix (GLDM), neighborhood gray-tone difference matrix (NGTDM), and wavelet-based features. These feature classes collectively captured a wide range of quantitative information related to intensity, shape, texture, and spatial relationships within the OCT images.

To ensure comparability and eliminate the influence of scale differences, all features were standardised by z-score transformation. The reproducibility of the extracted features was assessed using a subgroup of 20 randomly selected eyes to calculate the intraclass correlation coefficient (ICC). Features with ICC values > 0.75 were considered to have good reproducibility, and these features were further screened using the Mann-Whitney U test (*P* < 0.05). Subsequently, to further exclude the effect of anti-VEGF agent type on outcomes, dummy variables were created for categorical agent types. These screened OCT-omics features, alongside the dummy variables representing drug types, were input to logistic regression models using a recursive feature elimination approach, iteratively eliminating features with the smallest contributions to model performance.

### Development of OCT-omics models

The OCT-omics model was constructed in the training set, and OCT-omics scores were computed for each sample. The Mann-Whitney U test was utilised to compare OCT-omics scores between the PDME and non-persistent DME (NPDME) groups in both sets. Pearson correlation analysis examined the correlation between OCT-omics scores and CST change rate after treatment. Clinical utility was assessed using decision curve analysis (DCA) with net benefits quantified using the “rmda” R package.

### Development and validation of machine learning models

In this study, we developed and trained two machine learning models using the selected OCT-omics feature: a support vector machine (SVM) and a back propagation neural network (BPNN). The SVM model had a linear kernel function with penalty factor C set to 1 and a maximum of 1000 iterations. For the BPNN model, a single hidden layer with 30 neurons and the Rectified Linear Unit (ReLU) activation function were used, along with the Limited-memory Broyden–Fletcher–Goldfarb–Shanno (LBFGS) solver and an L2 regularisation term of 1. Both models were trained using five-fold cross-validation. The classification performances and generalisation abilities of three classifiers (logistic, SVM, and BPNN) were assessed in this study. Performance metrics, including accuracy ($\frac{\text{t}\text{r}\text{u}\text{e}\, \text{p}\text{o}\text{s}\text{i}\text{t}\text{i}\text{v}\text{e}\text{s}+\text{t}\text{r}\text{u}\text{e} \, \text{n}\text{e}\text{g}\text{a}\text{t}\text{i}\text{v}\text{e}\text{s}}{\text{t}\text{o}\text{t}\text{a}\text{l} \,\text{p}\text{u}\text{p}\text{u}\text{l}\text{a}\text{t}\text{i}\text{o}\text{n}}$), sensitivity (recall, $\frac{\text{t}\text{r}\text{u}\text{e}\, \text{p}\text{o}\text{s}\text{i}\text{t}\text{i}\text{v}\text{e}\text{s}}{\text{t}\text{r}\text{u}\text{e} \,\text{p}\text{o}\text{s}\text{i}\text{t}\text{i}\text{v}\text{e}\text{s}+\text{f}\text{a}\text{l}\text{s}\text{e} \,\text{n}\text{e}\text{g}\text{a}\text{t}\text{i}\text{v}\text{e}\text{s}}$), specificity ($\frac{\text{t}\text{r}\text{u}\text{e} \,\text{n}\text{e}\text{g}\text{a}\text{t}\text{i}\text{v}\text{e}\text{s}}{\text{t}\text{r}\text{u}\text{e}\, \text{n}\text{e}\text{g}\text{a}\text{t}\text{i}\text{v}\text{e}\text{s}+\text{f}\text{a}\text{l}\text{s}\text{e} \,\text{p}\text{o}\text{s}\text{i}\text{t}\text{i}\text{v}\text{e}\text{s}}$), positive predictive value (precision, $\frac{\text{t}\text{r}\text{u}\text{e}\, \text{p}\text{o}\text{s}\text{i}\text{t}\text{i}\text{v}\text{e}\text{s}}{\text{t}\text{r}\text{u}\text{e}\, \text{p}\text{o}\text{s}\text{i}\text{t}\text{i}\text{v}\text{e}\text{s}+\text{f}\text{a}\text{l}\text{s}\text{e}\, \text{p}\text{o}\text{s}\text{i}\text{t}\text{i}\text{v}\text{e}\text{s}}$), negative predictive value, ($\frac{\text{t}\text{r}\text{u}\text{e}\, \text{n}\text{e}\text{g}\text{a}\text{t}\text{i}\text{v}\text{e}\text{s}}{\text{t}\text{r}\text{u}\text{e} \,\text{n}\text{e}\text{g}\text{a}\text{t}\text{i}\text{v}\text{e}\text{s}+\text{f}\text{a}\text{l}\text{s}\text{e}\, \text{n}\text{e}\text{g}\text{a}\text{t}\text{i}\text{v}\text{e}\text{s}}$), and F1 score (2 $\times \frac{\text{p}\text{r}\text{e}\text{c}\text{i}\text{s}\text{i}\text{o}\text{n} \times \text{r}\text{e}\text{c}\text{a}\text{l}\text{l}}{\text{p}\text{r}\text{e}\text{c}\text{i}\text{s}\text{i}\text{o}\text{n}+ \text{r}\text{e}\text{c}\text{a}\text{l}\text{l}}$) were computed for both the training and test sets. Confusion matrices and ROC curves with corresponding AUC (area under the curve) values were visualised to assess classifier performance using the R packages “ggplot2”, “reshape2” and “pROC”.

### Statistical analysis

Statistical analyses were conducted using R software (version 4.1.1), Python (version 3.9.13), and the SPSSPRO online data analysis platform (https://www.spsspro.com). Continuous variables were described using means ± standard deviations (SD) or medians with interquartile ranges (IQR). Categorical variables were presented as counts and percentages. For comparisons between groups, independent t-tests or Mann-Whitney U tests were performed for continuous variables, depending on data normality. Chi-square or Fisher’s exact tests were used to examine associations between categorical variables. Pearson correlation analysis explored correlations between continuous variables. All tests were two-sided, with *P* < 0.05 considered statistically significant.

## Results

### Summary of study design and participant characteristics

**Fig. 1:**
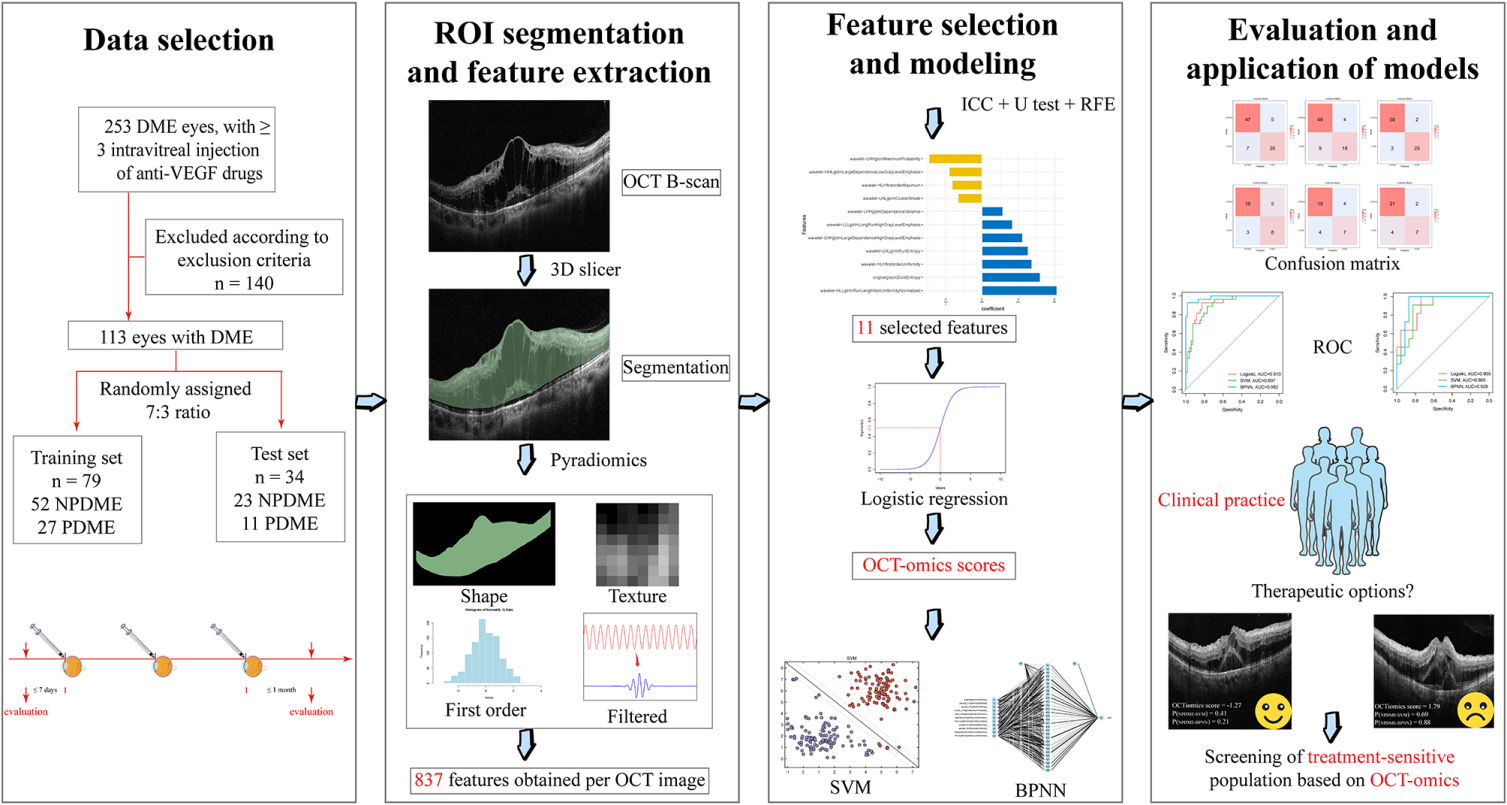
Flow chart of the study design

The study process involved a sequential flow, as depicted in Fig. 1. It encompassed the formulation of clinical concerns, screening of clinical data, acquisition of medical images, segmentation of regions of interest (ROI), feature engineering, model building, and evaluation [23].

Some of the elements in Fig. 1 (eyes, syringes, crowd) are from Servier Medical Art (http://smart.servier.com/), licensed under a Creative Common Attribution 3.0 Generic License.

A total of 113 eyes from 82 patients were included. Participant characteristics are summarised in Table 1. Among the 82 participants, 26 (31.71%) were women, 31 (37.80%) had both eyes included, mean age was 54 years (SD = 10), and mean glycated hemoglobin (HbA1c) level was 7.60% (SD = 1.28). Of the 113 eyes, 58 (51.33%) were right eyes. Mean initial CST was 478 μm (SD = 172), and mean interval between first and third treatments was 98.17 days (SD = 44.57). Table S1 illustrates the distribution of the initial three medication types (three individual drugs or a combination) among the total of 113 eyes, notably, no significant differences were observed, either between the training and test sets (*P* = 0.56) or within the NPDME and PDME subgroups (*P* = 0.64).

**Table 1:** Baseline demographic and clinical characteristics of the sample included

Characteristic		
Eye level		*N* = 113
Laterality	Right (%)	58 (51.33)
	Left (%)	55 (48.67)
Classification	PDME (%)	38 (33.63)
	NPDME (%)	75 (66.37)
CST, µm	Mean (SD)	478 (172)
	Median (IQR)	443 (205)
Interval between 1st and 3rd treatments, days	Mean (SD)	98.17 (44.57)
	Median (IQR)	76 (56)
Participant level		*N* = 82
Sex	Male (%)	56 (68.29)
	Female (%)	26 (31.71)
Age, years	Mean (SD)	54 (10)
	Median (IQR)	55 (11.00)
HbA1c, %	Mean (SD)	7.60 (1.28)
	Median (IQR)	7.35 (1.76)
Eye involvement	Unilateral (%)	51 (62.20)
	Bilateral (%)	31 (37.80)

Abbreviations: CST, central subfield thickness; HbA1c, glycated haemoglobin; IQR, interquartile range; NPDME, non-persistent diabetic macular edema; PDME, persistent diabetic macular edema; SD, standard deviation

### Development of an OCT-based radiomics classification model and its clinical implications

**Fig. 2:**
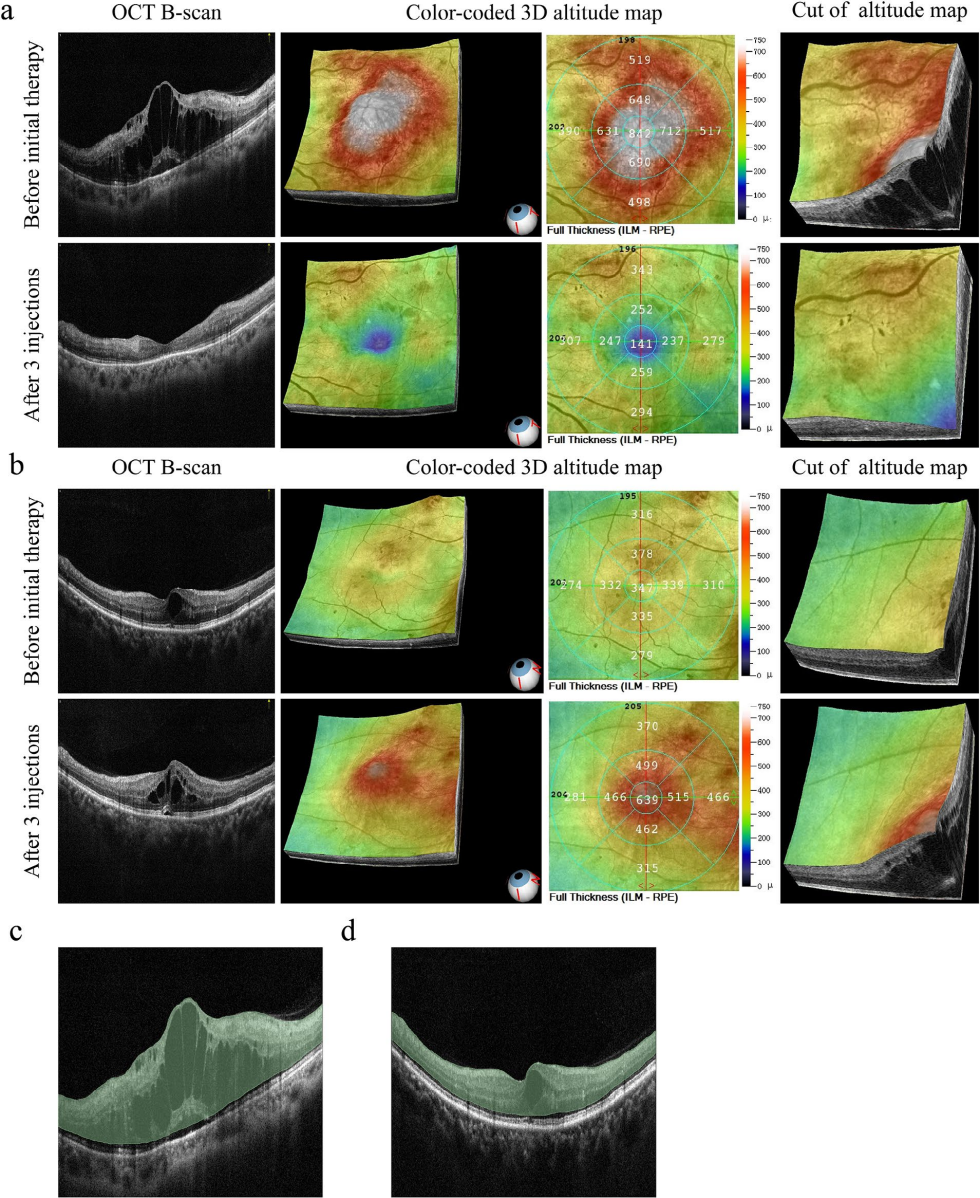
Images and ROI segmentation schematics of representative NPDME and PDME cases. (**a**) Representative NPDME case: 48-year-old woman with left eye involvement, initial CST of 842 μm, and baseline BCVA of counting fingers. After the receipt of three anti-VEGF treatments over 71 days, the CST substantially decreased to 141 μm and BCVA improved to 20/200. (**b**) Representative PDME case: 57-year-old man with right eye involvement, initial CST of 347 μm, and baseline BCVA of 20/40 (comparatively better than representative NPDME case). However, after the receipt of three anti-VEGF treatments over 90 days, the CST substantially increased to 639 μm and the BCVA decreased to 20/80. (**c**, **d**). Schematics illustrating the ROI segmentation procedure based on initial pretreatment OCT images for the two cases described above (**a** and **b**); the ROI comprises the region from the inner limiting membrane to the external limiting membrane

Among the 113 eyes, 75 had NPDME, and 38 had PDME. Figure 2 shows typical cases and OCT data from eyes with each type. 79 eyes were in the training set, and 34 in the test set.

**Fig. 3:**
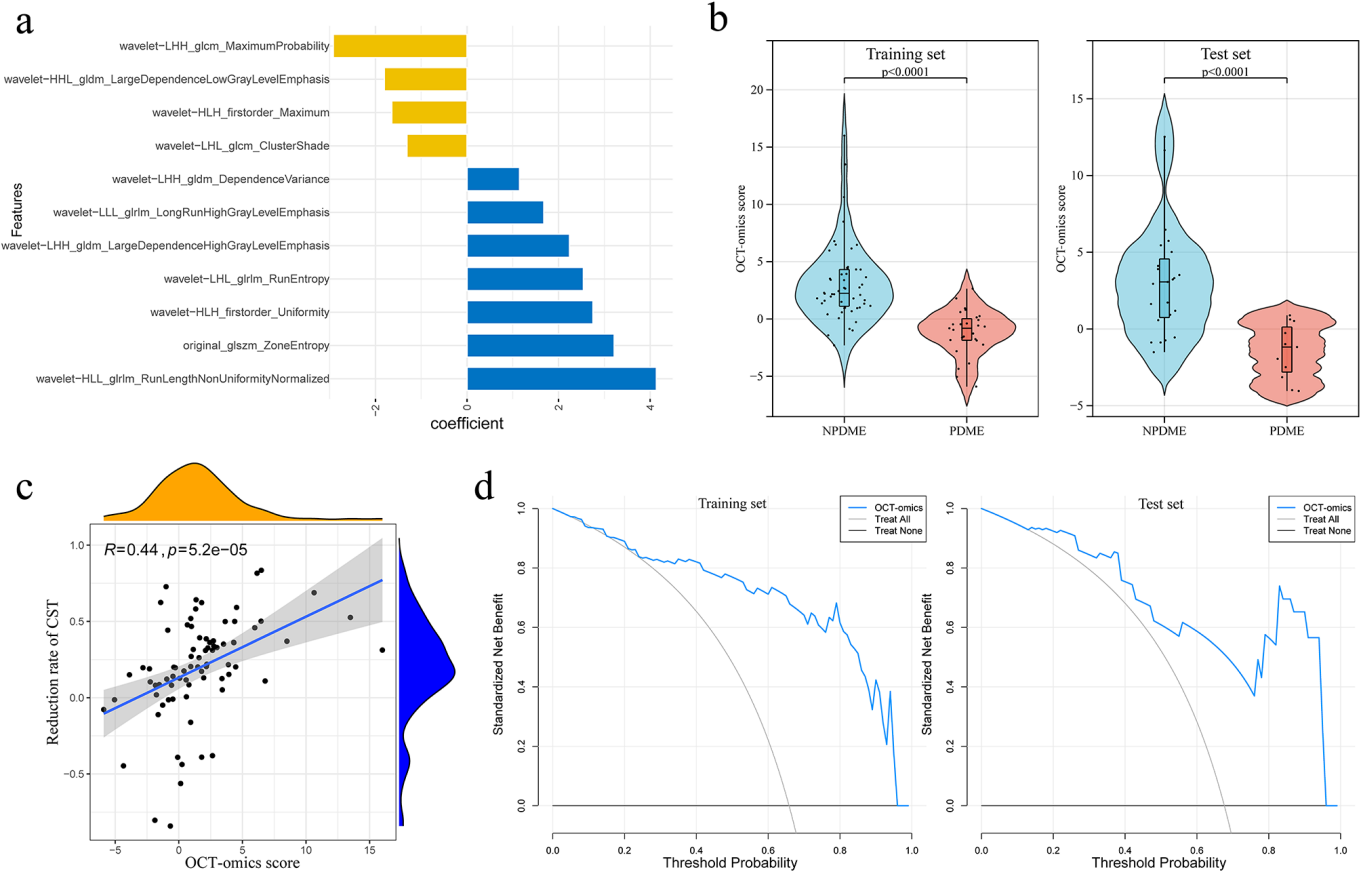
OCT-omics model evaluation and correlation analysis. (**a**) Coefficient distribution of the 11 features included in the logistic regression model. (**b**), Violin plots comparing OCT-omics scores between NPDME and PDME groups in the training and test sets. (**c**) Scatterplot showing the correlation between OCT-omics score and rate of CST reduction after treatment in the training set. (**d**) Decision curve analysis curves of the OCT-omics model in the training and test sets

In total 837 OCT-omics features were extracted from each eye. Within the training set, a screening process was conducted to refine the feature set. Initially, 775 features were screened according to ICC. Subsequently, 173 features were screened using the Mann-Whitney U test. From this combined set of selected features, along with the incorporation of 4 dummy variables representing drug types, a logistic regression model was constructed using the recursive feature elimination method (Table S2). The model included 11 key features, with the following equation: Y = 1.659 + 3.214 * original_glszm_ZoneEntropy – 1.313 * wavelet-LHL_glcm_ClusterShade + 2.542 * wavelet-LHL_glrlm_RunEntropy – 2.92 * wavelet-LHH_glcm_MaximumProbability + 1.146 * wavelet-LHH_gldm_DependenceVariance + 2.239 * wavelet-LHH_gldm_LargeDependenceHighGreyLevelEmphasis + 4.135 * wavelet-HLL_glrlm_RunLengthNonUniformityNormalised – 1.645 * wavelet-HLH_firstorder_Maximum + 2.751 * wavelet-HLH_firstorder_Uniformity – 1.806 * wavelet-HHL_gldm_LargeDependenceLowGreyLevelEmphasis + 1.677 * wavelet-LLL_glrlm_LongRunHighGreyLevelEmphasis (Fig. 3a). OCT-omics scores were significantly higher in the NPDME group than in the PDME group in both the training and test sets (*p* < 0.001) (Fig. 3b). OCT-omics scores positively correlated with CST reduction after treatment (*R* = 0.44, *p* < 0.001) (Fig. 3c). In both the training and test sets, DCA showed consistently superior net benefit compared with the “Treat none” strategy across all threshold probabilities and outperformed the “Treat all” strategy across a substantial portion of the threshold range, indicating that the OCT-omics score can provide a superior net benefit while minimising unnecessary treatment (Fig. 3d), indicating OCT-omics scores’ value in predicting treatment response in DME patients.

### Evaluation of overall performance in multiple models

The OCT-omics classification models (logistic, SVM, and BPNN) demonstrated strong discriminative abilities in both the training and test sets. In the training set, the logistic classifier achieved a sensitivity of 0.904 and a specificity of 0.741; the F1 score was 0.887, and the AUC was 0.910. The SVM classifier showed a sensitivity of 0.923 and a specificity of 0.667; the F1 score was 0.881 and the AUC was 0.897. The BPNN classifier exhibited a sensitivity of 0.962 and a specificity of 0.926; the F1 score was 0.962 and the AUC was 0.982. In the test set, the classifiers maintained their discriminative capabilities. The logistic classifier achieved a sensitivity of 0.783 and a specificity of 0.727, with an F1 score of 0.818 and an AUC of 0.905. The SVM classifier showed a sensitivity of 0.826 and a specificity of 0.636, with an F1 score of 0.826 and an AUC of 0.885. The BPNN classifier exhibited a sensitivity of 0.913 and a specificity of 0.636, with an F1 score of 0.875 and an AUC of 0.929.

**Fig. 4:**
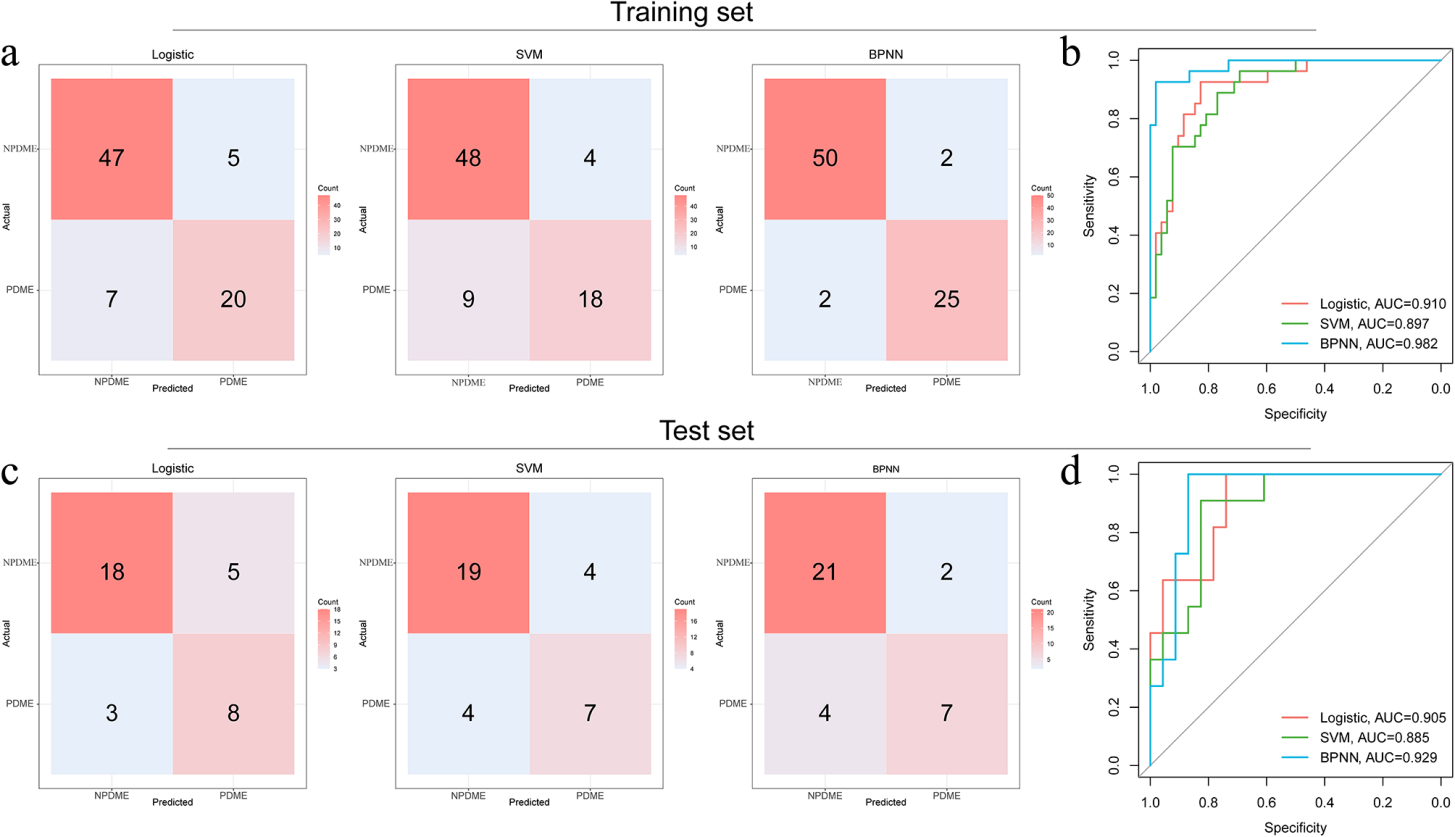
Classification performances of logistic, SVM, and BPNN models in training and test sets. (**a**). Confusion matrices for logistic, SVM, and BPNN models in the training set. (**b**) ROC curves for logistic, SVM, and BPNN models in the training set. (**c**) Confusion matrices for logistic, SVM, and BPNN models in the test set. (**d**) ROC curves for logistic, SVM, and BPNN models in the test set

Table 2 summarises the results, highlighting the robustness and generalisability of the developed OCT-omics classification models. High sensitivity values indicate efficacy in identifying positive cases (NPDME), whereas high specificity values indicate efficacy in identifying negative cases (PDME). F1 scores provide an overall assessment of model performance in terms of balancing specificity and sensitivity. The AUC values highlight discriminative power in distinguishing between PDME and NPDME subgroups according to OCT-omics features. Figure 4 visually represents the model performance through confusion matrices and ROC plots, providing an intuitive understanding of classifier performance and further supporting their discriminative ability.

In summary, the OCT-omics classification models exhibit robust performance and hold potential clinical implications. These findings establish OCT-omics as a non-invasive tool for predicting individualised treatment responses and assessing retinal pathologies, laying the foundation for future research in this area.

**Table 2:** Performances of predictive models in terms of treatment responses in patients with DME

		Training set			Test set	
	Logistic	SVM	BPNN	Logistic	SVM	BPNN
SEN	0.904	0.923	0.962	0.783	0.826	0.913
SPE	0.741	0.667	0.926	0.727	0.636	0.636
ACC	0.848	0.835	0.949	0.765	0.765	0.824
PPV	0.870	0.842	0.962	0.857	0.826	0.840
NPV	0.800	0.818	0.926	0.615	0.636	0.778
F1	0.887	0.881	0.962	0.818	0.826	0.875
AUC	0.910	0.897	0.982	0.905	0.885	0.929

SEN, sensitivity; SPE, specificity. ACC, accuracy; PPV, positive predictive value; NPV, negative predictive value; F1, F1 score; AUC, area under the receiver operating curve

### Clinical implications of the pretreatment OCT-omics prediction model

**Fig. 5:**
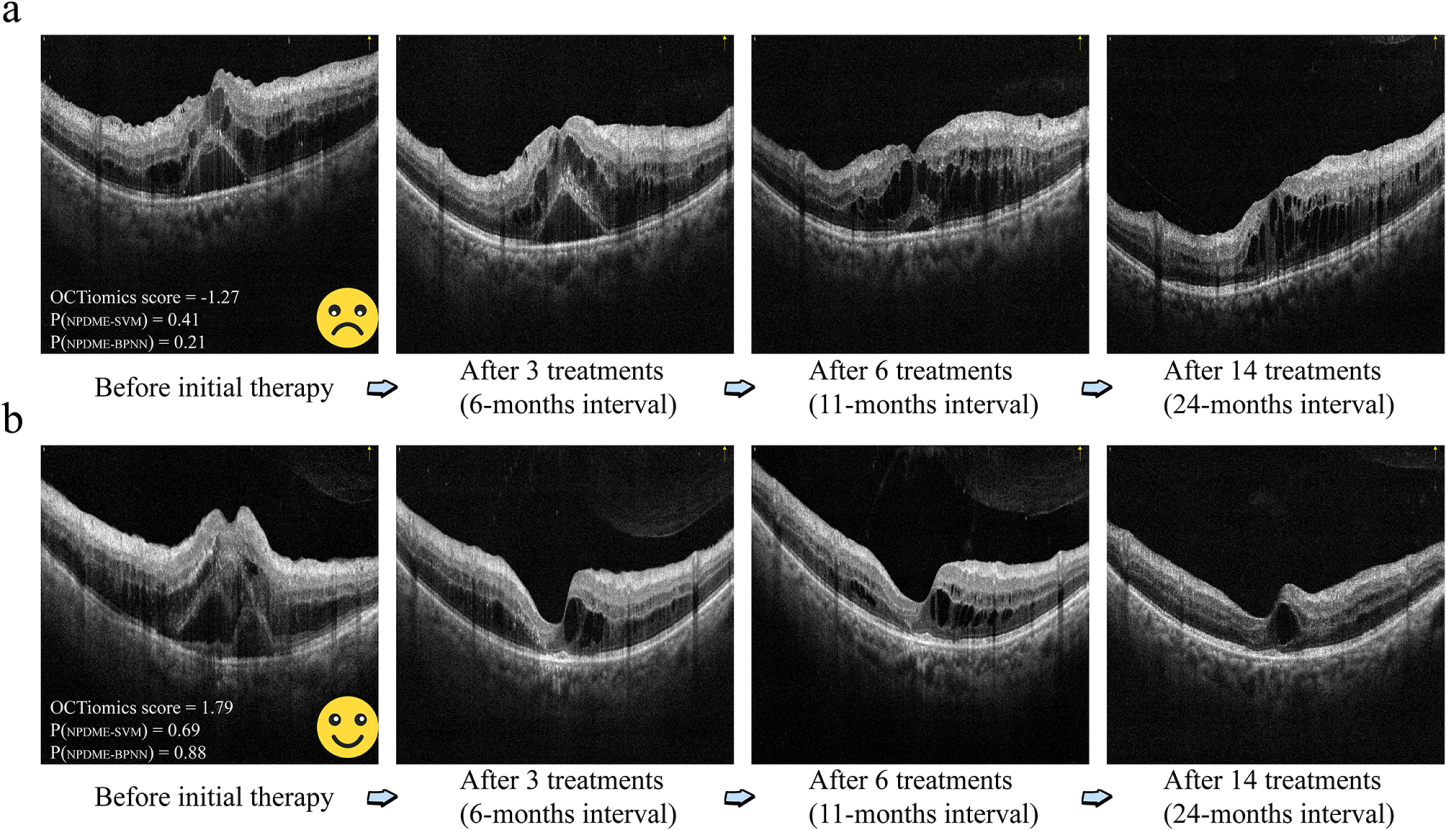
Retrospective follow-up of a patient with DME in both eyes after anti-VEGF treatment. (**a)** Long-term status of DME in the right eye. (**b**) Long-term status of DME in the left eye

Figure 5 illustrates the typical case with long-term anti-VEGF intravitreal treatment outcomes and predictions based on the OCT-omics model. The patient, a 55-year-old woman, underwent evaluation using the OCT-omics prediction model developed in this study.

Figure 5a shows the long-term status of the patient’s right eye. The initial CST was 697 μm, and the OCT-omics score assigned by the prediction model was − 1.27. The SVM model predicted a 41% probability for NPDME, while the BPNN model predicted a 21% probability for NPDME. Subsequent treatments did not lead to significant improvement in DME, as indicated by persistently high CST values: 731 μm after three treatments, 557 μm after six treatments, and 599 μm after 14 treatments. Figure 5b shows the long-term status of the patient’s left eye. The initial CST was 755 μm, and the OCT-omics score assigned by the prediction model was 1.79. The SVM model predicted a 69% probability for NPDME, while the BPNN model predicted an 88% probability for NPDME. This eye displayed a more favourable response to subsequent treatments; CST gradually decreased to 339 μm after three treatments, 274 μm after six treatments, and 317 μm after 14 treatments.

These retrospective findings highlight the predictive power of the pretreatment OCT-omics model in assessing the long-term prognosis of DME. The OCT-omics model provides valuable insights into potential treatment responses and disease progression in individual patients. It holds promise as a valuable tool for clinicians to make informed decisions and tailor treatment strategies for patients with DME.

### Discussion

In this study, we introduced the concept of OCT-omics, which combines OCT with radiomics analysis to improve the overall understanding of inner retinal layers in DME. By exploring the predictive capabilities of the OCT-omics classification model in assessing the response to anti-VEGF treatment in DME patients, it provides a comprehensive and quantitative evaluation of retinal pathology, offering a promising paradigm for precise and personalized management strategies.

The advent of anti-VEGF therapy significantly transformed the treatment landscape for DME. However, challenges such as safety concerns related to repeated injections, high costs, and variable treatment responses remain [8]. PDME rates ranging from 23% to 40% in various studies [24, 25]. In our study, PDME eyes constituted 33.63% of all cases, which is in line with previous reports. Due to the lack of a consensus on the definition and optimal management of PDME [14, 15], treatment strategies such as monthly or three loading doses, pro re nata (PRN), and treat and extend (T&E) are widely used [26, 27]. In our research, we explored PDME based on the completion of three anti-VEGF injections, consistent with current clinical practice. Additionally, we used the dynamic thresholds for PDME assessment based on the initial CST, providing a novel objective metric for evaluation of treatment response. These exploratory approaches will advance DME management and lay the foundation for further investigations. Previous studies have indicated that early initiation of anti-VEGF therapy and favorable treatment response are associated with better long-term outcomes [28]. Although our cross-sectional study did not include long-term follow-up data, we observed consistent treatment outcomes in some patients with long-term PDME who received anti-VEGF therapy. Further studies with larger sample sizes and longer follow-up periods will provide valuable insights into the long-term outcomes of patients with DME and the potential applications of our proposed method in clinical practice.

OCT has revolutionised DME management and research by providing a high-resolution, non-invasive, and rapid imaging modality [29]. Various OCT-based DME classification approaches, such as the “SAVE” classification [30] and “TCED-HFV” grading protocol [31] have been developed to quantitatively analyse changes in retinal structure, providing valuable insights for clinical decision-making. However, there remains a substantial gap in establishing consensus and guidelines for reliably predicting treatment outcomes using OCT parameters [32] Previous studies have explored various quantitative parameters, such as DRIL and ellipsoid zone/external limiting membrane integrity, as potential predictors of DME treatment response [14, 33]. However, most studies of DRIL have been qualitative (presence or absence) or semi-quantitative (measuring absolute length or proportion), limiting their predictive power [12]. In the present study, OCT-omics as a novel quantitative analysis approach that utilises deeper features from OCT images of the retina, facilitating the transition from visual interpretation to data-driven analysis. There is a need to establish standardised image processing and feature screening protocols, as well as more interpretable and clinically applicable OCT-omics models, to further advance the field of DME research and management.

Machine learning proves to be an effective tool in addressing complex classification problems encountered in the screening, diagnosis, and categorization of patients with DME [34]. Previous studies have predominantly focused on demographic indicators, laboratory tests, and established OCT parameters to predict DME treatment efficacy [35]. In contrast, the present study combines OCT-omics with machine learning, enabling the integration of comprehensive quantitative predictive indicators. The robustness of this approach is demonstrated by its performance evaluation metrics. Future research may involve further expansion of the training dataset, exploration of techniques such as automatic segmentation based on deep learning to improve feature extraction, and utilisation of other emerging technologies to enhance the precision and individualisation of DME management.

It is crucial to acknowledge the limitations of this study, particularly its retrospective nature and reliance on single-center data. The inherent biases such as selection bias, incomplete data, and potential confounding variables, can affect the generalizability of results to broader populations or diverse healthcare settings. However, despite these limitations, the study’s strengths, notably as the inaugural exploration of OCT-omics in assessing DME, offer significant insights into pioneering a novel research methodology for clinical decision-making. Moving forward, prospective studies encompassing larger cohorts and a comprehensive exploration of various ROI layers (outer retinal layer, whole retinal layer, choroidal layer) are imperative to validate and build upon these initial findings. On the basis of our proposed OCT-omics methodology, there is a pressing need for multicenter, large-sample, prospective studies with nuanced subgroup analyses at diverse levels, encompassing different demographics, medications, and other variables. These efforts are pivotal for steering towards a more evidence-based approach in understanding and managing DME.

### Conclusions

In conclusion, this study introduces a pioneering methodology, OCT-omics, which integrates OCT imaging with radiomics analysis to comprehensively assess DME. The developed radiomics model demonstrates promising predictive capabilities in evaluating treatment responses and propels future research paradigm for individualized DME management.

### Electronic supplementary material

Below is the link to the electronic supplementary material.

Supplementary Material 1

Supplementary Material 2

## Data Availability

The source data and R scripts used for analysis in this study are available upon reasonable request to the corresponding author.
