# Supplementary material for: Machine learning and optical coherence tomography-derived radiomics analysis to predict persistent diabetic macular edema in patients undergoing anti-VEGF intravitreal therapy
Source: J Transl Med. 2024 Apr 16;22:358. doi: 10.1186/s12967-024-05141-7 (PMC11022368; doi:10.1186/s12967-024-05141-7)
Supplement: Supplementary file 1 — Supplementary Material 1 [file 12967_2024_5141_MOESM1_ESM.docx]

|  | Dataset Division | | | DME Status | | |
| --- | --- | --- | --- | --- | --- | --- |
|  | Training Set | Test set | P | NPDME | PDME | P |
| Ranibizumab Alone  Aflibercept Alone | 12  23 | 7  6 | 0.56 | 11  21 | 8  8 | 0.64 |
| Conbercept Alone  Mixed | 26  18  79 | 11  10  34 |  | 23  20  75 | 14  8  38 |  |

**Table S1** Distribution of medication use in training/test sets and NPDME/PDME groups. The unit of count information in this table is eye.
